# Apulian infectious diseases network: survey on the prevalence of delta infection among chronic HBV carriers in Apulia

**DOI:** 10.3389/fpubh.2023.1247454

**Published:** 2023-09-26

**Authors:** Massimo Fasano, Michele Milella, Sergio Carbonara, Paolo Tundo, Salvatore Minniti, Giovanni Buccoliero, Anna Maria Maci, Sergio Lo Caputo, Teresa Antonia Santantonio

**Affiliations:** ^1^Infectious Diseases Unit, Ospedale della Murgia “F. Perinei”, Altamura, BA, Italy; ^2^Infectious Diseases Unit, Department of Biomedical Sciences and Human Oncology, University of Bari, Bari, Italy; ^3^Infectious Diseases Unit, Ospedale Vittorio Emanuele II, Bisceglie, BT, Italy; ^4^Infectious Diseases Unit, Ospedale S. Caterina Novella, Galatina, LE, Italy; ^5^Infectious Diseases Unit, Ospedale Perrino, Brindisi, Italy; ^6^Infectious Diseases Unit, Ospedale S. Giuseppe Moscati, Statte, TA, Italy; ^7^Infectious Diseases Unit, Ospedale Vito Fazzi, Lecce, Italy; ^8^Infectious Diseases Unit, Department of Medical and Surgical Sciences, University of Foggia, Foggia, Italy

**Keywords:** hepatitis B virus, Hepatitis Delta Virus, chronic viral hepatitis, hepatocellular carcinoma, epidemiology

## Abstract

**Background:**

The current prevalence and clinical burden of Hepatitis Delta Virus (HDV) infection in Apulia are unknown. This study aimed to define the current epidemiological scenario of delta infection and to detect difficulties in the diagnosis and clinical management of HDV patients in Apulia.

**Methods:**

From May to September 2022, a fact-finding survey was conducted at eight Infectious Diseases Units of the Apulian region; each Unit was asked to complete a questionnaire on screening and diagnosis of HDV infection and demographic, virological, and clinical characteristics of HDV patients.

**Results:**

A total of 1,461 HBsAg-positive subjects were followed up on an outpatient basis. Screening for HDV ranged from 30 to 90% of HBsAg + carriers in a single center. Overall, 952 HBsAg ± subjects (65%) were tested for HDV, and 80/952 (8.4%) were anti-HDV positive. Serum HDV RNA was detected only in 15/80 (19%) anti-HDV-positive subjects, and 12/15 patients (80%) were viremic. Sixty-five anti-HDV-positive subjects (81%) were from Italy; risk factors for HDV acquisition included the presence of HDV infection in the family (29/80 = 36%), drug addiction (12/80 = 15%), and co-infection with HCV or HIV (7/80 = 9%). Liver cirrhosis and hepatocellular carcinoma were diagnosed in 41 (51%) and 4 (5%) patients, respectively. Fifty-seven patients (71%) received nucleos(t)ide analog treatment.

**Conclusions:**

The results of this survey show that HDV screening is variable and insufficient, thus real prevalence data on delta infection are lacking in Apulia. Moreover, the HDV RNA test is not available in most laboratories and is not provided by the national health system. These results underline the need for an organizational model to optimize the management of HDV patients throughout the Apulian region.

## Introduction

Hepatitis Delta virus (HDV) is responsible for a severe form of chronic viral hepatitis that can rapidly progress to liver cirrhosis and hepatocellular carcinoma (HCC) at higher rates than in monoinfected chronic hepatitis B (HBV) carriers (1–3).

In Italy, the progressive reduction of HBV infection in recent decades, mainly due to mandatory anti-hepatitis B vaccination, has also reduced HDV circulation (4–7). However, despite the declining prevalence, chronic hepatitis delta, due to its severity, remains a major public health issue for the National Health System.

Considering the severity of the disease and in view of new therapeutic perspectives (8–10), it is crucial to early identify and treat patients with hepatitis delta and to acquire the epidemiological data necessary for appropriate social welfare planning.

To date, prevalence data on delta infection are lacking in Apulia. Here we report the results of a survey conducted among chronic HBV carriers followed at eight Infectious Diseases Units of the Apulian region in order to define the current epidemiological scenario of delta infection, to detect difficulties in the diagnosis, and to lay the foundations for an organizational model for the diagnosis and care of the patient with hepatitis Delta in Apulia.

## Methods

The Apulian Infectious Diseases Network includes eight Infectious Diseases Units distributed throughout the region. From May 2022 to September 2022, we carried out a Delta infection survey among all HBsAg carriers within the outpatient setting. Each unit was asked to complete a questionnaire containing the following information:

- The number of HBsAg-positive patients followed at each center
- The number of HBsAg-positive patients screened for HDV infection
- The number of HDV-positive patients
- The number of HDV-positive patients tested for serum levels of HDV RNA
- Risk factors for HDV infection
- Demographic characteristics of HDV-positive patients (age, sex, country of birth)
- Virological characteristics of HDV-positive patients (HBeAg/anti-HBe status, serum HBV DNA levels, serum HDV RNA levels, HDV genotype, coinfections with HCV and/or HIV)
- Clinical characteristics of HDV-positive patients (stage of liver fibrosis, presence of esophageal varices, diagnosis of HCC)
- Previous treatment with interferon
- Treatment with nucleos(t)ide analogs (NAs)

Each patient was given a progressive numerical code that included the province of the Center at which he or she was in follow-up. Virological and routine analyses were performed according to the best clinical practice of each clinical center. Anti-HDV, anti-HCV, and anti-HIV antibodies were tested by commercially available enzyme immunoassays. HDV-RNA was tested in two laboratories using an in-house PCR assay and more recently a commercially available assay (RoboGene HDV RNA quantification kit 2.0–lower limit of detection (LoD) 6 IU/ml. HDV genotype was assessed by genome sequencing in six patients with chronic delta hepatitis, enrolled in a clinical trial.

### Statistical analysis

Sociodemographic, clinical, and virological features of the study population were collected and presented in terms of the number of subjects and percentages for categorical variables and of the mean (±Standard Deviation, SD) or median (Inter Quartile Range, IQR) for continuous variables in accordance with their parametric of non-parametric distribution. A *p*-value < 0.05 was considered statistically significant. Analysis was performed using the Jamovi package 2.3.2.

## Results

**Figure 1:**
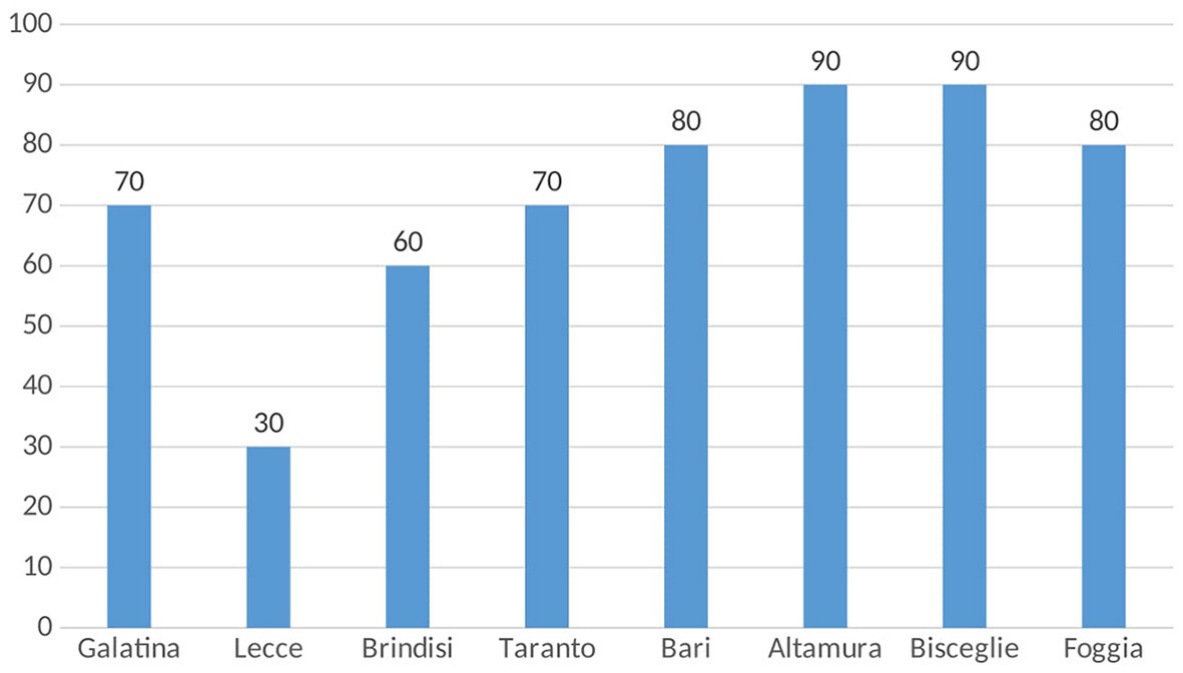
Percentages of HBsAg-positive patients screened for HDV infection across eight Apulian infectious disease units.

A total of 1,461 HBsAg-positive subjects were followed up on an outpatient basis at the eight Infectious Diseases Units in Apulia. Screening for HDV was highly variable, varying from 30 to 90% of HBsAg+ carriers at a single center (Figure 1). Of the 1,461 HBsAg ± patients, only 952 subjects (65%) were tested for HDV and 80 of them (8.4%) were anti-HDV-positive.

**Figure 2:**
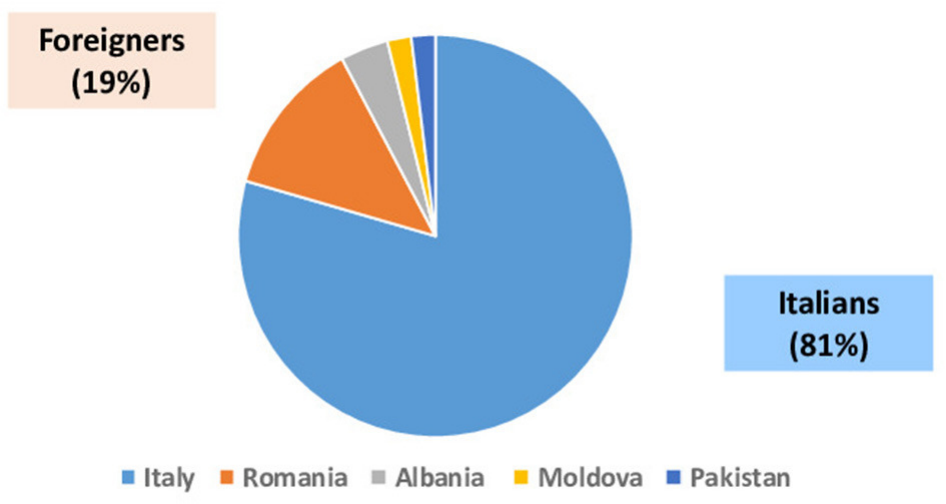
Country of origin of 80 HBsAg+/anti-HDV+ patients.

Sixty-five anti-HDV-positive subjects (81%) were from Italy, while the remaining 15 (19%) were foreigners, mainly from Eastern Europe such as Romania, Albania, and Moldova (Figure 2).

Demographic, virological, and clinical characteristics of the 80 subjects with delta infection are shown in the Table 1. Fifty-four (68%) were men, and the median age was 58 years (range 28–85). Only 15 patients (19%) were tested for HDV RNA in two Apulian laboratories using an in-house PCR assay and more recently a commercially available quantitative assay; 12/15 patients were viremic: 4/7 were tested by a home-made PCR assay and 8/8 were tested using the commercial kit. In these latter patients, median serum HDV-RNA levels were 154,365.50 IU/ml (range 4,840–5,804,510 UI/ml). HDV genotype was available for six patients with chronic hepatitis D enrolled in a clinical trial, who were infected by genotype 1.

**Table 1:** Demographic, virological, and clinical features of 80 HBsAg+/anti-HDV+ patients.

	**Overall(*N* = 80)**	**Italians (*N* = 65)**	**Foreigners (15)**	***p***
Sex, male (%)	50 (68)	46 (71)	8 (53)	*ns*
Age, years, median (range 28–85)	58	57	43	*ns*
**Risk factors**				
Intrafamiliar spread, *n (%)*	29 (36)	27 (42)	2 (13)	*ns*
Drug use, *n (%)*	12 (15)	12 (18)	0	
HIV infection, *n (%)*	2 (3)	2 (3)	0	
HCV infection, *n (%)*	5 (6)	5 (8)	0	
**Tested for HDV RNA**, ***n (%)***	15 (19)	11 (17)	4 (27)	*ns*
HDV RNA positive*, n (%)*	12 (80)	9 (82)	3 (75)	
HBV DNA positive, *n (%)*	18 (23)	13 (20)	5 (33)	*ns*
HBeAg positive, *n (%)*	2 (3)	0	2 (13)	*ns*
Anti-HBe positive, *n (%)*	78 (97)	65 (100)	0	*ns*
Cirrhosis, *n (%)*	41 (51)	34 (52)	7 (47)	*ns*
HCC, *n (%)*	4 (5)	4 (6)	0	*ns*
**Therapy**				
- NAs, *n (%)*	57 (71)	46 (71)	11 (73)	*ns*
- IFN, *n (%)*	33 (41)	26 (40)	7 (47)	

N, number; HBV, Hepatitis B virus; HDV, Hepatitis D virus; HCV, Hepatitis C virus; HIV, Human Immunodeficiency Virus; NA, Nucleos(t)ide analog; IFN, Interferon; HCC, Hepatocellular carcinoma.

All 80 HBV/HDV patients were tested for HBV DNA, and detectable levels were found in 18/80 (23%) subjects. HBeAg positivity was found in only two patients of foreign nationality.

Among the risk factors for delta virus acquisition, the most frequent was the presence of HDV infection in the family (29 patients = 36%), other factors were drug addiction (12 patients = 15%), co-infection with HCV (five patients = 6%), or HIV (two patients = 3%). Liver cirrhosis was diagnosed in 41 patients (51%), and HCC development was reported in four patients (5%).

Thirty-three patients (41%) received one or more cycles of IFN without virological response and 57 (71%) were on treatment with NAs.

Data for both Italian and foreign subjects are shown in the Table 1. No significant differences were observed or described between the two groups.

## Discussion

In this survey conducted among HBsAg-positive subjects followed at eight Apulian Infectious Disease Units, the anti-HDV prevalence was 8.4%. This prevalence rate is similar to that recently reported in other Italian studies among HBsAg carriers with liver disease of varying severity followed in specialized centers (4–7). In a nationwide survey, Stroffolini and colleagues reported an anti-HDV overall prevalence of 9.9% (6.4% in Italian natives and 26.4% in non-natives) among 786 chronic HBsAg carriers consecutively referring to 9 tertiary centers in Italy (5). Moreover, data from the nationwide longitudinal PITER HBV/HDV ongoing cohort, reported an overall anti-HDV prevalence of 9.2% among 3,679 HBsAg carriers in care by 50 clinical centers (6, 7).

One of the main critical issues that emerged from the survey is that screening for HDV is highly variable and completely insufficient in some centers, due to the COVID-19 pandemic and possibly to the low awareness among healthcare providers of the persistent clinical and economic impact of delta hepatitis. The prevalence of HDV infection parallels the severity of liver disease and is higher in patients with liver cirrhosis or hepatocellular carcinoma (1). This survey does not provide the prevalence of delta infection among cirrhotic patients, however, the presence of cirrhosis in more than half of the patients with HBV/HDV infection confirms the severity of this double infection and the relevant pathogenic role of HDV.

Therefore, it is a priority to encourage the early identification of delta-infected individuals by implementing screening for HDV in all HBsAg-positive individuals with automatic laboratory detection of anti-HDV antibodies in all samples tested positive for HBsAg (reflex testing).

Another critical issue that emerged from the survey was the difficulty in accessing HDV RNA testing. The test is essential for documenting the presence of active delta infection and monitoring the effectiveness of therapy; however, it is not available in most laboratories and is not reimbursed by the national health system. According to national and international guidelines, HDV RNA should be quantified using well-standardized, validated real-time PCR assays (11).^1^ Given that quantitative determination of HDV RNA is a fundamental requirement for an accurate diagnosis of ongoing HDV infection and to monitor antiviral treatment, identifying reference laboratories equipped to perform delta viremia is crucial (12).

Lastly, less than half of the patients performed one or more cycles of IFN therapy without benefit. In these patients, the new drugs will serve to broaden treatment options.

In conclusion, the results of this survey conducted among chronic HBV carriers followed at eight Infectious Diseases Units of the Apulian region, show that real HDV prevalence data in Apulia are lacking and that HDV screening is variable and insufficient. Moreover, the HDV RNA test is not available in most laboratories and is not provided by the national health system. These data underline the need for an organizational model to implement screening strategies, facilitate diagnosis, and optimize the management of HDV patients throughout the Apulia region.

## Data availability statement

The original contributions presented in the study are included in the article/supplementary material, further inquiries can be directed to the corresponding author.

## Author contributions

All authors listed have made a substantial, direct, and intellectual contribution to the work and approved it for publication.

## Footnotes

^1^ https://www.webaisf.org/2023/03/14/indicazioni-operativeaisf-e-simit-per-la-diagnosi-e-la-gestione-clinica-del-paziente-affetto-da-epatitedelta/
